# Arterial stiffness during whole‐body passive heat stress in healthy older adults

**DOI:** 10.14814/phy2.14094

**Published:** 2019-05-07

**Authors:** Zachary J. Schlader, Yoshiyuki Okada, Stuart A. Best, Qi Fu, Craig G. Crandall

**Affiliations:** ^1^ Center for Research and Education in Special Environments Department of Exercise and Nutrition Sciences University at Buffalo Buffalo New York; ^2^ Institute for Exercise and Environmental Medicine Texas Health Presbyterian Hospital Dallas University of Texas Southwestern Medical Center Dallas Texas; ^3^ Department of Special Care Dentistry Hiroshima University Hiroshima Japan; ^4^ Department of Kinesiology and Health Promotion University of Kentucky Lexington Kentucky

**Keywords:** Aging, arterial compliance, central blood pressure, hyperthermia, pulse wave velocity, wave reflection

## Abstract

We tested the hypothesis that whole‐body passive heat stress reduces arterial stiffness in older adults. At preheat stress (baseline) and when core temperature was elevated by 0.6 ± 0.2°C (mild) and 1.2 ± 0.3°C (moderate), arterial stiffness was measured in eight healthy younger (26 ± 5 years) and eight healthy older (70 ± 4 years) adults in the supine position. Arterial stiffness was estimated from carotid‐to‐femoral pulse wave velocity (cfPWV, applanation tonometry). cfPWV was higher at baseline in older adults (8.8 ± 2.3 m/sec vs. 5.6 ± 0.9 m/sec, *P* < 0.01) and this difference was maintained throughout passive heat stress (*P* < 0.01). cfPWV did not change (*P* ≥ 0.49) with passive heat stress in either younger (at moderate heat stress: 6.0 ± 1.0 m/sec) or older (at moderate heat stress: 8.5 ± 1.6 m/sec) adults. However, the influence of baseline cfPWV on the change in cfPWV during mild (*r* = −0.66, *P* = 0.04) and moderate (*r* = −0.87, *P* < 0.01) heat stress were inversely related in older adults, and the strength of these relations was not statistically different (*P* = 0.08). In younger adults, the influence of baseline cfPWV on the change in cfPWV during mild heat stress was also inversely related (*r* = −0.79, *P* = 0.01), while the strength of this relation was attenuated at moderate heat stress (*r* = −0.24, *P* = 0.30). Changes in arterial stiffness during passive heat stress in adults aged ≥65 year are likely dependent on the magnitude of baseline arterial stiffness and not necessarily age.

## Introduction

Whole‐body passive (i.e., resting) heat stress reduces total peripheral resistance and elevates cardiac output (Wilson and Crandall 2011). These responses are caused primarily by cutaneous vasodilation, which occurs in proportion to increases in core and skin temperatures (Wilson and Crandall 2011). This increased skin blood flow results in an increased shear stress through large conduit arteries (Naylor et al. 2011; Thomas et al. 2016b). Shear stress promotes the endogenous production of vasodilators (e.g., nitric oxide) that increase arterial elasticity and reduce arterial stiffness (Kinlay et al. 2001; Bellien et al. 2010). As a result, passive heat stress is often found to acutely reduce vascular stiffness in younger individuals (Caldwell et al. 2017). However, the magnitude of these reductions is often dependent on baseline arterial stiffness before passive heat stress. For instance, those subjects with high baseline arterial stiffness demonstrate reductions in arterial stiffness during heat stress. Whereas those with low baseline arterial stiffness often do not demonstrate any changes in arterial stiffness during heat stress (Ganio et al. 2012; Moyen et al. 2016).

Aging is associated with large artery stiffening, which is caused by the development of fibrosis and collagen cross‐linked products in the arterial walls (Lee and Oh 2010). Thus, even healthy older adults generally have stiffer arteries compared to healthy younger adults (Vaitkevicius et al. 1993). Whole‐body passive heat stress, invoked by Finnish sauna bathing (i.e., ~30 min intermittent exposure to a ~73°C, 10–20% relative humidity environment), acutely reduces arterial stiffness following exposure in middle‐aged adults (mean age: 52 years) (Lee et al. 2018). To our knowledge, it remains unknown if these findings are consistent in healthy older adults (defined as age ≥65 years) – a group of people at a greater risk of cardiovascular disease compared to middle‐aged adults – if these responses differ between younger and older adults, and if there is a dose–response relationship between the magnitude of the increase in body temperature and changes in arterial stiffness. Drawing from the aforementioned findings in younger individuals (Ganio et al. 2012; Moyen et al. 2016), elevations in baseline arterial stiffness suggest that arterial stiffness may be reduced during passive heat stress in an older population. On the other hand, the cardiovascular responses to whole‐body passive heat stress are often attenuated in older adults, such that the reductions in total peripheral resistance, and increases in cardiac output and skin blood flow are attenuated (Minson et al. 1998; Greaney et al. 2015; Gagnon et al. 2017). This indirectly suggests that the increase in shear stress during passive heat stress may be lower in older adults, which may attenuate passive heat stress invoked reductions in arterial stiffness in this population, compared to younger adults. That said, recent evidence suggests that shear stress may not be differentially affected by heat stress in older adults given that conduit artery shear rate during pronounced heating of the lower limbs (i.e., skin temperature ~42°C for 30+ min) did not differ between healthy older and younger adults (Romero et al. 2016).

Collectively, the available literature suggests that arterial stiffness may be either unaffected (due to an attenuated shear stress response) or reduced (due to elevated baseline arterial stiffness) during whole‐body passive heat stress in older adults. Therefore, the purpose of this study was to determine the effect of progressive whole‐body passive heat stress on arterial stiffness in older adults. Based on previous findings (Ganio et al. 2012; Moyen et al. 2016; Lee et al. 2018), we hypothesized that whole‐body passive heat stress progressively reduces arterial stiffness in older adults.

## Methods

### Ethical approval

The study and informed consent were approved by the Institutional Review Board at the University of Texas Southwestern Medical Center in Dallas and Texas Health Presbyterian Hospital Dallas. The study conformed to the standards set by the Declaration of Helsinki, except for registration in a database. Before completing any study related activities, each subject was fully informed of the experimental procedures and possible risks before giving informed, written consent.

### Subjects

Eight healthy younger (three females) and nine healthy older (four females) subjects participated in this study. The subject characteristics are listed in Table 1. All subjects were nonsmokers, not taking medications (except oral contraceptives), and reported to be free from any known cardiovascular, metabolic, or neurological diseases. Younger females were not pregnant, which was confirmed via a urine pregnancy test. The menstrual cycle phase was not controlled. This was deemed acceptable because all experimental testing was held on the same day and arterial stiffness is not affected by menstrual cycle phase (Priest et al. 2018). Older females were postmenopausal and not currently taking hormone replacement therapy. Subjects arrived at the laboratory euhydrated (confirmed via a urine specific gravity <1.028, Table 1) and having refrained from food for at least 2 h and strenuous exercise, alcohol, and caffeine for a period of 24 h.

**Table 1:** Subject characteristics

	Younger	Older	*P*‐value
Male/Female	5/3	5/4	–
Age (y)	26 ± 5	70 ± 4	<0.01
Height (cm)	174 ± 12	169 ± 10	0.42
Weight (kg)	72.0 ± 15.3	71.5 ± 9.0	0.94
Body mass index (kg/m^2^)	23.6 ± 3.2	24.9 ± 2.3	0.35
Body surface area (m^2^)	1.9 ± 0.2	1.8 ± 0.2	0.73
Urine specific gravity	1.015 ± 0.010	1.014 ± 0.006	0.91

Mean ± SD.

### Instrumentation and measurements

Body temperature was controlled via a water‐perfused tube lined suit (Med‐Eng, Ottawa, ON, Canada) that covered the entire body except the head, hands, and feet. Approximately 90 min prior to experimental testing, each subject swallowed a telemetry pill (HQ Inc., Palmetto, FL, USA), which provides a reliable measure of core body temperature during prolonged passive heating (Pearson et al. 2012). Mean skin temperature was measured under the water‐perfused suit as the weighted average of six thermocouples (Omega Engineering, Inc. Stamford, CT, USA) attached to the following locations: abdomen (14%), calf (11%), chest (22%), lower back (19%), thigh (14%), and upper back (20%) (Taylor et al. 1989). Heart rate was continually recorded from an electrocardiogram (HP Patient Monitor, Agilent, Santa Clara, CA, USA) interfaced with a cardiotachometer (CWE, Ardmore, PA). Nude body weight was measured with a scale (Health‐o‐meter Professional Scales, McCook, IL), and pre‐ to post‐ trial changes in nude body weight provided an index of changes in body fluid loss.

At each measurement period, arterial blood pressure was measured via auscultation of the brachial artery by electrosphygmomanometry (Tango+, SunTech, Raleigh, NC) and mean arterial pressure was calculated as diastolic pressure plus 1/3 pulse pressure. Carotid‐to‐femoral pulse wave velocity (cfPWV), the gold standard measure of central arterial stiffness (Laurent et al. 2006), was measured using applanation tonometry (SphygmoCor, AtCor Medical, Sydney, Australia) by sequentially recording electrocardiogram gated carotid and femoral artery pressure waveforms (Butlin and Qasem 2016). Transit distance between the carotid and femoral artery measurement locations was calculated as the surface distance from the suprasternal notch to the carotid and femoral recording sites. Pulse wave velocity could not be accurately measured in one older female subject. Thus, these data are presented as *n* = 8. As a secondary analysis, a central aortic pressure waveform was constructed from pulse waves obtained at the radial artery using tonometry and a validated generalized transfer function (Pauca et al. 2001). The radial artery waveform was calibrated from brachial artery electrosphygmomanometric measurements of systolic and diastolic pressure. Aortic pulse pressure was calculated as the difference between the diastolic and systolic aortic pressures. Indices of pulse wave reflection were also calculated from the synthesized aortic pressure waveforms. These measures are often interpreted as indicators of the overall systemic arterial stiffness and changes in vascular resistance downstream of the aorta (Butlin and Qasem 2016). Aortic augmentation pressure was calculated as the difference between the first aortic systolic pressure peak and the reflected wave. Aortic augmentation pressure was also expressed as a percentage of the aortic pulse pressure (termed the augmentation index). Heart rate was simultaneously determined from the aortic waveform and was used to standardize the augmentation index for a heart rate of 75 bpm. This was deemed necessary because: (1) heart rate is known to independently affect the augmentation index (Wilkinson et al. 2000), (2) passive heat stress will increase heart rate (Wilson and Crandall 2011), and (3) the increase in heart rate during passive heat stress will likely be attenuated in older adults (Gagnon et al. 2017). Finally, the time delay of the reflected wave was calculated as the time difference between the beginning of systole to the arrival of the reflected wave. In all instances, the applanation tonometry measurement locations were marked with indelible ink to ensure that the measurement locations were the same at all measurement periods. All applanation tonometry measurements were made by the same operator (YO) and are presented as the average of two consistent high quality measurements at each time point. Measurement quality was determined based on the standards placed forth by the SphygmoCor software.

Skin blood flow was measured via laser Doppler flowmetry (Perimed, Stockhold, Sweden) on the ventral surface of the lower leg (half way between the knee and ankle) under the water‐perfused suit. Skin blood flow was measured at this location because it was distal to the femoral applanation tonometry measurement location. At the end of the experimental protocol, the area surrounding the laser Doppler probe was heated to 44°C for 30 min to produce a local heating induced skin blood flow maximum. Cutaneous vascular conductance (CVC) was calculated as laser Doppler flux divided by brachial mean arterial pressure. Skin blood flow and CVC data are presented as a percentage of the local heating maximal response. Notably, the maximal skin blood flow (younger: 91 ± 33 PU, older: 99 ± 84 PU, *P* = 0.80) and CVC (younger: 1.00 ± 0.39 PU/mmHg, older: 1.01 ± 0.73 PU, *P* = 0.98) responses did not differ between younger and older adults. Due to technical problems, skin blood flow was not measured in two older subjects. Thus, the skin blood flow and CVC data in the older group are presented as *n* = 7.

### Experimental protocol

Following instrumentation, subjects assumed the supine position in a temperature‐controlled laboratory (22–24°C) while 34°C water perfused the suit. Following at least 20 min rest, baseline hemodynamic measurements were taken. Subjects were then passively heated by circulating ~49°C water through the suit. Hemodynamic measurements were then obtained when core temperature increased by 0.6 ± 0.2°C (mild heat stress) and again when core temperature increased 1.2 ± 0.3°C (moderate heat stress). Heating time to mild heat stress was 34 ± 8 min in younger adults and 35 ± 6 min in older adults, which did differ between groups (*P* = 0.68). Heating time from mild to moderate heat stress was 14 ± 3 min in younger adults and 18 ± 8 min in older adults, which also did not differ between groups (*P* = 0.22). Thus, the time course of measurement periods did not differ between groups. Following the final series of hemodynamic measurements, 20°C water was then circulated through the water‐perfused suit, which promoted the recovery of body temperature back towards baseline levels. Subjects were not allowed to drink at any time during the experimental procedures.

### Data and statistical analyses

Heart rate and thermoregulatory data were sampled continuously at 50 Hz via a data acquisition system (Biopac MP150, Goleta, CA, USA). Heart rate and thermoregulatory data are presented as a 60 sec average immediately before obtaining the tonometry data. Subject characteristics between groups were compared using independent sample *t*‐tests. All other data were analyzed using a 2‐way mixed model ANOVA with one between‐ (group) and one within‐ (heat stress level) subject factors. When the ANOVA revealed a significant *F* test, post hoc Sidak adjusted pairwise comparisons were made. Linear relations between baseline cfPWV and the change in cfPWV during passive heat stress was assessed in younger and older adults at mild (i.e., the differences between baseline and mild heat stress) and moderate (i.e., the differences between baseline and moderate heat stress) heat stress using Pearson produce moment correlation analysis, as has been employed previously (Ganio et al. 2012; Moyen et al. 2016). Changes in the r values between mild and moderate heat stress within each group were compared via the methods of Meng et al. (1992), permitting comparison of changes in the relative strength of the examined relations. Using pooled data, linear relations between baseline cfPWV and the change in cfPWV during mild and moderate heating were also examined, which may provide insights regarding whether the aforementioned correlations are dependent on baseline cfPWV rather than age. Whole‐body passive heat stress often reduces blood pressure (Crandall and Wilson 2014), and changes in blood pressure can independently influence cfPWV (Townsend et al. 2015). To explore this possibility, we also examined the linear relation between changes in mean arterial pressure and changes in cfPWV during passive heat stress in both younger and older adults using Pearson produce moment correlation analysis. All data were analyzed using Prism software (Version 6, GraphPad Software Inc. La Jolla, CA). A priori statistical significance was set at *P* ≤ 0.05 and actual *P*‐values are reported where possible. Data are reported as mean ± SD.

## Results

### Thermoregulatory responses

In both younger and older adults, core temperature increased from baseline to mild and to moderate heat stress (all *P* < 0.01), with no differences between groups (*P* = 0.37, Table 2). Mean skin temperature also progressively increased from baseline to the mild heat stress testing period (*P* < 0.01) in both groups (Table 2). However, the increase in mean skin temperature at the mild heat stress was greater in older adults (older adults: +4.4 ± 0.7°C vs. younger adults: +3.5 ± 0.7°C, *P* = 0.01), but this difference was not apparent at the moderate heat stress (*P* = 0.31, Table 2). Skin blood flow and CVC also increased with progressive whole‐body passive heat stress (*P* < 0.01), but the magnitude of these increases was not different between groups (*P* ≥ 0.64, Table 2). Total sweat loss was greater in the younger adults (younger adults: 0.7 ± 0.2 L vs. older adults: 0.4 ± 0.3 L, *P* = 0.06), which represented a greater percentage loss of body weight in younger adults (younger adults: 1.0 ± 0.2% vs. older adults: 0.6 ± 0.4%, *P* = 0.03).

**Table 2:** Thermoregulatory responses to passive heat stress

	Baseline	Mild	Moderate	2‐way ANOVA		
				Group	Heat	Group × Heat
Core temperature (°C)						
Younger (*n* = 8)	36.9 ± 0.1	37.5 ± 0.2^B^	38.2 ± 0.4^B^^,^ M	0.37	<0.01	0.05
Older (*n* = 9)	36.9 ± 0.3	37.4 ± 0.3^B^	37.9 ± 0.3^B^^,^ M			
Mean skin temperature (°C)						
Younger (*n* = 8)	34.4 ± 0.6	37.9 ± 0.4^B^	38.4 ± 0.5^B^	0.02	<0.01	0.05
Older (*n* = 9)	34.1 ± 0.6	38.6 ± 0.4^B^^,^ *	38.8 ± 0.5^B^			
Skin blood flow (% max)						
Younger (*n* = 8)	14 ± 7	47 ± 16^B^	52 ± 17^B^	0.87	<0.01	0.93
Older (*n* = 7)	13 ± 7	44 ± 17^B^	52 ± 15^B^			
CVC (% max)						
Younger (*n* = 8)	16 ± 8	54 ± 18 B	63 ± 20 B	0.64	<0.01	0.60
Older (*n* = 7)	16 ± 7	47 ± 13 B	62 ± 18 B ^,^ M			

Mean ± SD, *indicates different from Younger (*P* = 0.02), ^B^indicates different from Baseline (*P* < 0.01), ^M^indicates different from Mild (*P* = 0.04).

### Central and peripheral hemodynamics

Heart rate increased with progressive whole‐body passive heat stress in both groups (*P* < 0.01, Table 3). However, this increase was attenuated in older adults at mild (older adults: +18 ± 4 bpm vs. younger adults: 29 ± 8 bpm, *P* < 0.01) and moderate (older adults: +23 ± 5 bpm vs. younger adults: 38 ± 11 bpm, *P* < 0.01) heat stress (Table 3). Brachial systolic, diastolic and mean arterial pressures did not change with progressive whole‐body passive heat stress (*P* ≥ 0.22) and there were no differences between groups (*P* ≥ 0.22, Table 3). Brachial artery pulse pressure also did not differ between younger and older adults (*P* = 0.62, Table 3). However, brachial pulse pressure increased from baseline to mild heat stress in younger adults by 4 ± 6 mmHg (*P* < 0.01), but was unchanged from baseline in older adults at this stage of passive heat stress (*P* = 0.96, Table 3). Aortic systolic and pulse pressures were higher at baseline in the older adults (*P* < 0.01, Table 3). Aortic systolic pressure and pulse pressures did not change with progressive whole‐body passive heat stress in younger adults (*P* ≥ 0.67, Table 3). However, aortic systolic pressure decreased at mild heat stress in older adults (*P* = 0.02), and aortic pulse pressure was lower than baseline at both mild (*P* = 0.01) and moderate (*P* < 0.01) heat stress in older adults (Table 3).

**Table 3:** Central and peripheral hemodynamic responses to passive heat stress

	Baseline	Mild	Moderate	2‐way ANOVA		
				Group	Heat	Group × Heat
Heart rate (bpm)						
Younger (*n* = 8)	62 ± 13	91 ± 12^B^	100 ± 8^B^^,^ M	0.07	<0.01	<0.01
Older (*n* = 9)	61 ± 12	79 ± 12^B^	84 ± 12^B^,*			
Brachial						
Systolic pressure (mmHg)						
Younger (*n* = 8)	114 ± 17	115 ± 11	115 ± 8	0.23	0.56	0.41
Older (*n* = 9)	124 ± 6	119 ± 8	116 ± 21			
Diastolic pressure (mmHg)						
Younger (*n* = 8)	64 ± 8	61 ± 12	56 ± 7	0.04	0.07	0.98
Older (*n* = 9)	70 ± 7	68 ± 9	62 ± 14			
Mean pressure (mmHg)						
Younger (*n* = 8)	80 ± 7	79 ± 11	75 ± 7	0.06	0.45	0.93
Older (*n* = 9)	88 ± 5	85 ± 8	80 ± 16			
Pulse pressure (mmHg)						
Younger (*n* = 8)	50 ± 6	54 ± 3^B^	60 ± 5	0.62	0.35	0.01
Older (*n* = 9)	55 ± 10	51 ± 7	54 ± 11			
Aortic						
Systolic pressure (mmHg)						
Younger (*n* = 8)	98 ± 7	97 ± 12	93 ± 7	<0.01	0.04	0.41
Older (*n* = 9)	116 ± 8*	107 ± 7^B^	103 ± 19			
Diastolic pressure (mmHg)						
Younger (*n* = 8)	65 ± 8	63 ± 12	58 ± 7	0.06	0.13	0.98
Older (*n* = 9)	70 ± 7	69 ± 9	64 ± 14			
Mean pressure (mmHg)						
Younger (*n* = 8)	79 ± 8	78 ± 12	74 ± 7	0.01	0.17	0.94
Older (*n* = 9)	89 ± 5	86 ± 8	82 ± 16			
Pulse pressure (mmHg)						
Younger (*n* = 8)	33 ± 3	34 ± 3	35 ± 4	0.04	0.07	0.01
Older (*n* = 9)	46 ± 10*	38 ± 6^B^	36 ± 12^B^			

Mean ± SD, *indicates different from Younger (*P* ≤ 0.01), B indicates different from Baseline (*P* ≤ 0.05). M indicates different from Mild (*P* < 0.01).

### Indices of arterial stiffness

**Figure 1:**
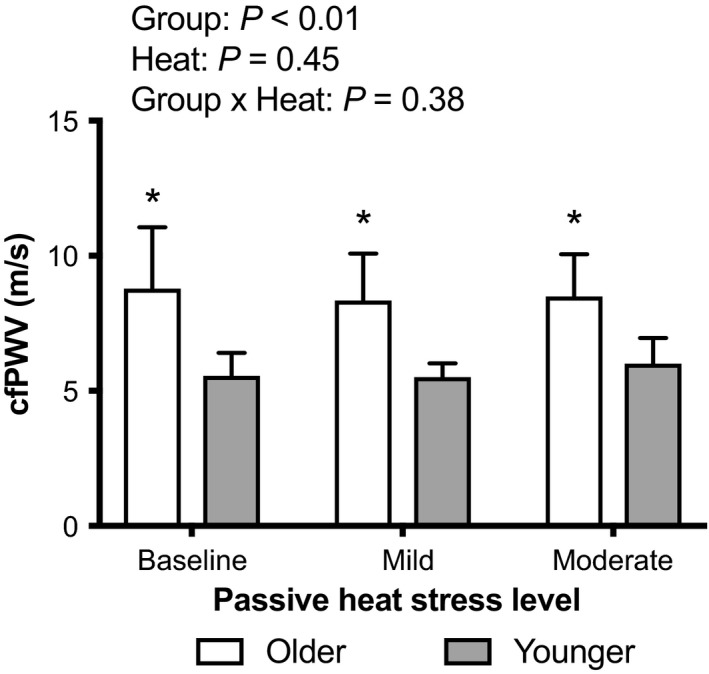
Carotid‐to‐femoral pulse wave velocity (cfPWV) during passive heat stress in Younger (*n* = 8) and Older (*n* = 8) adults. Mean ± SD. * indicates different from Younger (*P* < 0.01). *P*‐values for two‐way mixed‐model ANOVA are noted.

Carotid‐to‐femoral pulse wave velocity was higher at baseline in older adults (*P* < 0.01) and this difference was maintained throughout progressive whole‐body passive heat stress (*P* < 0.01, Fig. 1). Both mild and moderate passive heat stress did not affect cfPWV in either younger or older adults (*P* ≥ 0.49, Fig. 1). At mild heat stress, the change in cfPWV from baseline was −0.4 ± 1.7 m/sec in older adults and −0.1 ± 0.6 m/sec in younger adults, while at moderate heat stress the change in cfPWV from baseline was −0.3 ± 0.9 m/sec in older adults and 0.4 ± 0.7 m/sec in younger adults. There were no group (main effect: *P* = 0.30), heat stress (main effect: *P* = 0.45), or group × heat stress (interaction: *P* = 0.38) differences. Notably, changes in mean arterial pressure and changes in cfPWV during passive heat stress in both younger (*r* = 0.09, *P* = 0.76) and older (*r* = −0.25, *P* = 0.35) adults were not correlated, suggesting that changes in blood pressure did not have an independent influence on the observed changes in cfPWV.

**Figure 2:**
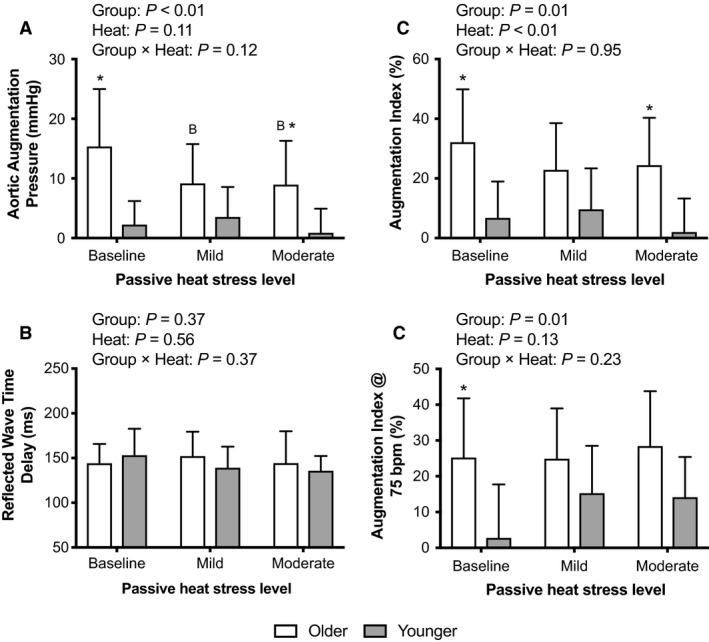
Aortic augmentation pressure (A), reflected wave time delay (B), the augmentation index (C) and the augmentation index at a heart rate of 75 bpm (D), indices of pulse wave reflection, during passive heat stress in Younger (*n* = 8) and Older (*n* = 9) adults. Mean ± SD. * indicates different from Younger (*P* ≤ 0.04), ^B^ indicates different from Baseline (*P* = 0.05). *P*‐values for two‐way mixed‐model ANOVA are noted.

Aortic augmentation pressure was elevated at baseline and moderate passive heat stress in older adults (*P* < 0.01, Fig. 2A). Aortic augmentation pressure did not change with progressive whole‐body passive heat stress in younger adults (*P* ≥ 0.68), but was lower than baseline at both mild (*P* = 0.05) and moderate (*P* = 0.04) passive heat stress in older adults (Fig. 2A). The reflective wave time delay did not differ between younger and older adults (*P* ≥ 0.73) and was unaffected by progressive passive heat stress (*P* ≥ 0.39, Fig. 2B). The augmentation index was higher at baseline and moderate passive heat stress in older adults compared to younger adults (*P* < 0.01), but was unaffected by progressive passive heat stress in either group (*P* ≥ 0.24, Fig. 2C). The augmentation index corrected for a heart rate of 75 bpm demonstrated a similar trajectory (Fig. 2D); the exception was that there were statistically significant differences between younger and older adults at baseline (*P* < 0.01), but not at mild (*P* = 0.44) or moderate (*P* = 0.13) heat stress (Fig. 2D).

**Figure 3:**
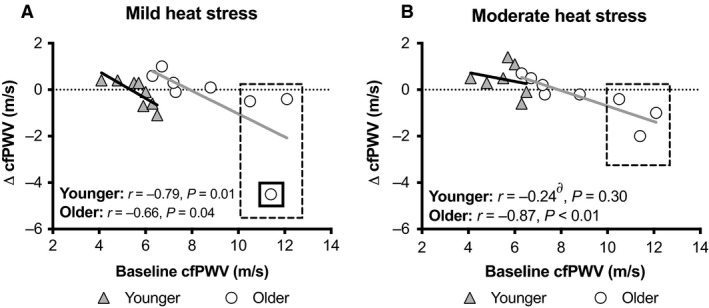
Relations between baseline carotid‐to‐femoral pulse wave velocity (cfPWV) and the change (Δ) in cfPWV during mild (A) and moderate (B) passive heat stress in Younger (*n* = 8) and Older (*n* = 8) adults. ∂ indicates different from mild heat stress (*P* < 0.01). Dashed line square highlights the three older adults with baseline cfPWV >10 cm/sec who also demonstrated the greatest reductions in cfPWV with passive heat stress. Although there is no reason to believe it is an outlier, it is notable that the removal of the data point with a solid square around it in the mild heat stress figure (A) did not meaningfully change the results (*r* = −0.82, *P* = 0.01). Notably, similar findings were found when the data obtained from the younger and older adults were pooled together at mild (*r* = −0.56, *P* = 0.03) and moderate (*r *= −0.77, *P* < 0.01) heat stress.

The influence of baseline cfPWV on the subsequent change in cfPWV during passive heat stress was significantly, negatively related in both younger and older at mild heat stress (Fig. 3A). At moderate heat stress, these variables remained significantly negatively related in older adults, but not younger adults (Fig. 3B). In younger adults, baseline cfPWV explained more variance in the change in cfPWV at mild heat stress compared to moderate heat stress (*P* < 0.01, Fig. 3). However, in older adults the relative strength of the relation between baseline cfPWV and the change in cfPWV did not statistically differ between mild and moderate heat stress (*P* = 0.08, Fig. 3). Notably, these relations did not meaningfully change when the data obtained from the younger and older adults were pooled together at mild (*r* = −0.56, *P* = 0.03) and moderate (*r* = −0.77, *P* < 0.01) heat stress.

## Discussion

This study tested the hypothesis that whole‐body passive heat stress reduces arterial stiffness in older adults aged ≥65 y. In contrast to this hypothesis, our data indicate that progressive passive heat stress did not affect cfPWV in older adults (Fig. 1). That said, we did identify a relatively strong negative relation between baseline cfPWV and the change in cfPWV during both mild and moderate passive heat stress in older adults (Fig. 3). As such, these findings suggest that changes in arterial stiffness during passive heat stress in older adults may be related to baseline arterial stiffness and not age per se.

### Arterial stiffness during passive heat stress in older adults

It is well established that arterial stiffness increases with advancing age (Lee and Oh 2010). Indeed, we observed that older adults presented with higher baseline cfPWV compared to younger adults (Fig. 1). The effect of passive heat stress on arterial stiffness is likely dependent on preheat stress arterial stiffness. For instance, in younger individuals those presenting with stiffer arteries at baseline demonstrate reductions in arterial stiffness with passive heat stress, whereas those with relatively compliant arteries preheat stress demonstrate little change in arterial stiffness during heat stress (Ganio et al. 2012; Moyen et al. 2016). Indeed, arterial stiffness is reduced immediately following Finnish sauna bathing in middle‐aged adults with an average baseline cfPWV of ~10 m/sec (Lee et al. 2018). With this background, it was surprising that, as a group, the older adults in our study did not demonstrate reductions in arterial stiffness with passive heat stress (Fig. 1). One explanation for these apparently diverging responses is that the cardiovascular responses during graded passive heat stress in the supine position is likely different than those occurring immediately following Finnish sauna bathing, which is characterized by two 15 min exposures to a very hot environment (~73°C, 10–20% relative humidity environment) in the seated position that is interspersed with a 2 min shower. These differences may be related to differential changes in body temperatures caused by differences in the heat stress modality. Unfortunately, however, Lee et al. (2018) did not report changes in core or skin temperatures. Another explanation is that the older adults in our study were healthy and had, on average, a cfPWV within healthy ranges, which is often defined as a cfPWV of <10 m/sec (Van Bortel et al. 2012). That said, we did find that baseline cfPWV was inversely related to changes in cfPWV during both mild and moderate passive heat stress in older adults (Fig. 3). This observation may be explained by a subset of three older adults with baseline cfPWV exceeding 10 m/sec (dashed box in Fig. 3). Indeed, a post hoc comparison of the absolute changes in cfPWV during passive heat stress in these three older adults with those of the five older adults with cfPWV <10 m/sec suggests that the older adults with stiffer arteries at baseline demonstrated greater reductions in cfPWV with passive heat stress (average change in cfPWV: −1.5 ± 1.5 cm/sec vs. 0.3 ± 0.4 cm/sec, *P* = 0.04). Collectively, these findings suggest that changes in arterial stiffness during passive heat stress in older adults are likely due to baseline arterial stiffness and not necessarily biological age. It is worth mentioning, however, that this conclusion is supported by data obtained from only three older adults. Thus, further studies are required to formally discern the role of baseline arterial stiffness on passive heat stress evoked changes in arterial stiffness in older adults. Nevertheless, these findings support a growing body of literature that indicates that the impact of whole‐body passive heat stress on arterial stiffness is dependent on baseline arterial stiffness (Ganio et al. 2012; Moyen et al. 2016).

### Central blood pressure and pulse wave reflection during passive heat stress in older adults

As a supplement to the cfPWV data, we also examined measures of aortic blood pressure and indices of pulse wave reflection. In the present study, passive heat stress acutely reduced aortic systolic pressure and aortic pulse pressure in the older adults (Table 3). To our knowledge, these observations in older adults are unique. However, our data support previous findings that Finnish sauna bathing reduces aortic pulse pressure for up to 30 min following the exposure in a group of adults with an average age of 52 years (Lee et al. 2018). Importantly, elevations in aortic pulse pressure are independently associated with adverse cardiovascular outcomes (Roman et al. 2009). Thus, that passive heat stress acutely reduces aortic pulse pressure in older adults may have clinical ramifications.

We also found that aortic augmentation pressure was reduced with passive heat stress in older adults (Fig. 2A). These changes were not likely caused by a shorter delay in the arrival of the reflected wave during passive heat stress because the reflected wave time delay was unaffected by whole‐body passive heat stress in either group (Fig. 2B). The augmentation index data suggest that the acute reductions in aortic augmentation pressure in older adults may be related to the acute reductions in aortic pulse pressure (Table 3). Specifically, the augmentation index did not change with passive heat stress in older adults (Fig. 2C and D). Augmentation index presents aortic augmentation pressure as a function of aortic pulse pressure (Butlin and Qasem 2016). Thus, the observed reductions in aortic augmentation pressure with passive heat stress in older adults likely occurred in proportion to reductions in aortic pulse pressure. Reductions in pulse wave reflection are often considered to confer cardiovascular benefits (Vlachopoulos et al. 2010a). However, this is typically manifested as a reduction in indices of the augmentation index. Thus, the potential importance of the observed changes in aortic augmentation pressure in the absence of a change in augmentation index is unclear.

### Potential mechanisms by which heat stress may promote cardiovascular benefits in individuals with elevated arterial stiffness

Arterial stiffness is a function of anatomical and physiological factors. Anatomical factors include the structural elements of the arteries (e.g., collagen content, etc.). A single bout of passive heat stress is unlikely to acutely induce structural remodeling. Thus, there are a few physiological mechanisms by which passive heat stress may promote acute reductions in arterial stiffness in individuals with stiffer arteries. First, whole‐body passive heat stress increases blood flow in conduit vessels (Wilson and Crandall 2011). The resulting increase in shear stress (Naylor et al. 2011; Thomas et al. 2016b) promotes endogenous production of vasodilators (e.g., nitric oxide), which are known to increase arterial elasticity and reduce arterial stiffness (Kinlay et al. 2001; Bellien et al. 2010). Thus, it may be that the magnitude of shear stress during passive heat stress is greater in those individuals with stiffer arteries, which could augment endogenous vasodilator production. We did not measure conduit artery shear stress in the present study. However, recent evidence indicates that conduit artery shear rate to a maximal heating stimulus (skin temperature ≈42°C) to the lower limbs does not differ between younger and older adults (Romero et al. 2016). Thus, we believe it is unlikely that conduit artery shear stress during passive heat stress differed between younger and older adults in the current study. However, it is possible that comparatively smaller heating stimulus in the present study (skin temperature ≈38°C) resulted in a differential conduit artery shear stress profile between younger and older adults. Nevertheless, it is also feasible that shear stress during passive heat stress was greater in those older adults with elevated baseline arterial stiffness. It is also possible that the sensitivity to (or the production of) endogenous vasodilators during passive heat stress was enhanced in those older adults with elevated baseline arterial stiffness. However, such a possibility is unlikely based on observations that conditions associated with elevated arterial stiffness (e.g., hypertension) are often associated with a reduced endogenous vasodilator reactivity and/or bioavailability (Smith et al. 2011).

Second, sympathetic activation independently increases arterial stiffness (Swierblewska et al. 2010). We did not measure indices of sympathetic activation in the current study. That said, it is well established that passive heat stress increases sympathetic activation in a dose–dependent manner, as evidenced by increases in muscle sympathetic nerve activity that occur in parallel with increases in core temperature (Low et al. 2011). The increase in muscle sympathetic nerve activity during passive heat stress does not differ between older and younger adults (Gagnon et al. 2015). Thus, it is unlikely that the magnitude of increases in sympathetic activation differed between the older and younger adults in the present study. However, it may be that the increase in sympathetic activation with passive heat stress was attenuated in those older adults with elevated baseline arterial stiffness or that those with stiffer arteries have a blunted responsiveness to sympathetic activation. To our knowledge, the independent effect of arterial stiffness on sympathetic activation during passive heat stress is unknown. However, it is possible that differential sympathetic activation and/or end organ responsiveness contributes to the reductions in arterial stiffness during passive heat stress observed in those individuals with elevated baseline arterial stiffness.

Third, acute reductions in blood pressure can reduce arterial stiffness (Stewart et al. 2003). Brachial artery blood pressures did not change with passive heat stress and did not differ between younger and older adults (Table 3). Moreover, changes in mean arterial pressure were not related to changes in cfPWV in either older or younger adults. However, aortic systolic pressure decreased with passive heat stress in older adults (Table 3). Thus, it is possible that acute reductions in aortic systolic pressure might partially explain reductions in cfPWV in older adults with high baseline arterial stiffness. However, the extent of the contribution of these changes in aortic systolic pressure to remains uncertain.

An interesting observation was that baseline cfPWV explained more variance in the change in cfPWV at mild heat stress compared to moderate heat stress in younger adults, but not in older adults (Fig. 3). This may suggest that there is a nonlinear dose–response relationship between the extent of increases in skin and/or core temperatures and the magnitude of reductions in arterial stiffness, and that this dose–response relationship may differ between younger and older adults. Thus, it may be that there is an optimal dose of passive heat stress sufficient to induce reductions in arterial stiffness, and that this dose may depend on the magnitude of baseline arterial stiffness and/or biological age. However, this remains to be experimentally investigated. Possible mechanism(s) underlying potential dose–response relationships may be due to differences in conduit artery shear stress profiles, sympathetic activation and/or responsiveness, and/or blood pressure changes evoked by progressive elevations in body temperature. Clearly, further research is required to uncover the physiological mechanisms by which passive heat stress promotes acute reductions in arterial stiffness, particularly in those individuals with elevated baseline arterial stiffness.

### Considerations

A few methodological considerations warrant mentioning. First, the relationship between pulse wave velocity and arterial stiffness is only valid when blood viscosity does not change (Bramwell and Hill 1922). Thus, a change in blood viscosity could change arterial stiffness independent of pulse wave velocity. We did not measure blood viscosity. Thus, in the present study we do not know if blood viscosity acutely changed in the younger or older adults. Increases in blood temperature to ~39.5°C reduces blood viscosity by ~10% (Çinar et al. 2001), while increases in hematocrit, as occurs with sweat loss and the subsequent reduction in plasma volume, increases blood viscosity (Pries et al. 1992). The magnitude of the increase in core temperature during passive heat stress did not differ between the younger and older adults in the present study (Table 2). However, the magnitude of body fluid losses was greater in the younger adults. Thus, it is possible that blood viscosity differed between younger and older adults during the latter stages of passive heat stress. Second, increases in heart rate can independently increase arterial stiffness (Lantelme et al. 2002), which likely occurs subsequent to a shortened artery recoil time (Mangoni et al. 1996). The increase in heart rate during passive heat stress was attenuated in older adults (Table 3). Thus, it is possible that reductions in arterial stiffness in the younger adults were masked by the higher heart rates in this group. Third, we did not control for menstrual cycle phase in our younger female subjects. Menstrual cycle hormones modulate the mechanisms underlying blood pressure regulation (Wenner and Stachenfeld 2012) and blood pressure regulation during heat stress is likely different between males and females (Meendering et al. 2005). Thus, the central hemodynamic responses to passive heat stress might be modulated by menstrual cycle phase. Fourth, it should be acknowledged that, to our knowledge, the generalized transfer function used to derive the aortic pressure waveform from radial tonometry has not been validated during passive heat stress. Finally, we recruited a relatively low number of participants in both our younger and older groups. This may have limited our ability to identify statistically significant differences throughout whole‐body passive heat stress and between groups. In the interest of full disclosure, therefore, we have presented these data as mean ± SD and have reported actual p‐values throughout the manuscript. We believe this best allows the reader to independently interpret these data.

### Perspectives

Aging is associated with large artery stiffening (Lee and Oh 2010). Central artery stiffening is associated with an increased risk of cardiovascular morbidity and mortality (Vlachopoulos et al. 2010b). Thus, interventions aiming to prevent or reverse age‐related increases in arterial stiffness have clinical ramifications. One such intervention is heat therapy, whereby people are repeatedly subjected to passive local (e.g., leg) (Romero et al. 2016; Thomas et al. 2016a) or whole‐body (Brunt et al. 2016) heat stress. Eight weeks of whole‐body heat stress reduces arterial stiffness in young healthy sedentary adults (Brunt et al. 2016). To our knowledge, the effects of chronic passive heat stress on arterial stiffness in older adults are unknown. That said, this study presents important information regarding the acute effects of passive heat stress on arterial stiffness in older adults. Specifically, we found that baseline arterial stiffness was inversely related to reductions in arterial stiffness invoked by passive heat stress. Notably, those people with elevated arterial stiffness prior to undergoing passive heat stress are those considered most at risk of cardiovascular events (Vlachopoulos et al. 2010b). Thus, our study provides some evidence that passive heat stress might be a therapeutic strategy for acutely reducing arterial stiffness in older adults presenting with elevated arterial stiffness.

## Conclusions

The findings of the present study indicate that progressive whole‐body passive heat stress does not affect arterial stiffness in groups of older and younger adults. However, we did identify that the relation between baseline arterial stiffness and the change in arterial stiffness during passive heat stress was negatively related in older adults. These findings suggest that changes in arterial stiffness during passive heat stress in older adults may be dependent on the magnitude of baseline arterial stiffness and not necessarily biological age.

## Conflict of Interest

There are no conflicts of interest to report.
